# The Probiotic *Lactobacillus rhamnosus* for Alleviation of *Helicobacter pylori*-Associated Gastric Pathology in East Africa

**DOI:** 10.3389/fmicb.2018.01873

**Published:** 2018-08-14

**Authors:** Nieke Westerik, Gregor Reid, Wilbert Sybesma, Remco Kort

**Affiliations:** ^1^Department of Molecular Cell Biology, VU University Amsterdam, Amsterdam, Netherlands; ^2^Yoba for Life Foundation, Amsterdam, Netherlands; ^3^Canadian R&D Centre for Human Microbiome and Probiotics, Lawson Health Research Institute, London, ON, Canada; ^4^Department of Microbiology and Immunology, and Surgery, Western University, London, ON, Canada; ^5^Department of Microbiology and Systems Biology, Netherlands Organization for Applied Scientific Research, Zeist, Netherlands; ^6^ARTIS-Micropia, Amsterdam, Netherlands

**Keywords:** *Lactobacillus rhamnosus* yoba 2012, *Lactobacillus rhamnosus* GG, *Helicobacter pylori*, gastric pathology, ulcer, eradication therapy, Uganda, East Africa

## Abstract

The probiotic *Lactobacillus rhamnosus* GG (LGG) can play a role in establishing a harmless relationship with *Helicobacter pylori* and reduce gastric pathology in East African populations. *H. pylori* has the ability to inhabit the surface of the mucous layer of the human stomach and duodenum. In the developing world, an estimated 51% of the population is carrier of *H. pylori*, while in some Western countries these numbers dropped below 20%, which is probably associated with improved sanitation and smaller family sizes. Colonization by *H. pylori* can be followed by inflammation of the gastric mucus layer, and is a risk factor in the development of atrophic gastritis, peptic ulcers and gastric cancer. Notwithstanding the higher prevalence of *H. pylori* carriers in developing countries, no equal overall increase in gastric pathology is found. This has been attributed to a less pro-inflammatory immune response to *H. pylori* in African compared to Caucasian populations. In addition, a relatively low exposure to other risk factors in certain African populations may play a role, including the use of non-steroidal anti-inflammatory drugs, smoking, and diets without certain protective factors. A novel approach to the reduction of *H. pylori* associated gastric pathology is found in the administration of the probiotic bacterium *Lactobacillus rhamnosus* yoba 2012 (LRY), the generic variant of LGG. This gastro-intestinal isolate inhibits *H. pylori* by competition for substrate and binding sites as well as production of antimicrobial compounds such as lactic acid. In addition, it attenuates the host’s *H. pylori*-induced apoptosis and inflammation responses and stimulates angiogenesis in the gastric and duodenal epithelium. The probiotic LRY is not able to eradicate *H. pylori* completely, but its co-supplementation in antibiotic eradication therapy has been shown to relieve side effects of this therapy. In Uganda, unlike other African countries, gastric pathology is relatively common, presumably resulting from the lack of dietary protective factors in the traditional diet. Supplementation with LRY through local production of probiotic yogurt, could be a solution to establish a harmless relationship with *H. pylori* and reduce gastric pathology and subsequent eradication therapy treatment.

## Introduction

During the last decade of our probiotic yogurt programs in the East-African countries Uganda, Tanzania, and Kenya, we took note of the high incidence of self-reported ulcers and accompanying stomach pains, as reported decades ago for in particular Northern Tanzania and South-Western Uganda and some regions in Kenya (Tovey and Tunstall, 1975; Balint, 1998). Interestingly, during our fieldwork aimed at the stimulation of local production and consumption of probiotic yogurt to improve health and wealth in local communities, we observed a decrease of complaints among consumers and realized that there might be an effect from the intake of probiotic yogurt containing *Lactobacillus rhamnosus* yoba 2012 (LRY), the generic variant of *Lactobacillus rhamnosus* GG (LGG) (Kort and Sybesma, 2012; Sybesma et al., 2013), on the reduction of symptoms associated with ulcers.

Over the last years a number of reviews have been published on *H. pylori* ulcers (Blaser and Atherton, 2004; Cover and Blaser, 2009), the prevalence of *H. pylori* and ulcers in different populations (Kidd et al., 1999b; Roberts et al., 2016), the interaction between *H. pylori* and LGG (Hamilton-Miller, 2003; Gotteland et al., 2006), and effects of LGG administration on ulcers (Lam et al., 2007a,b). The present paper is, however, the first review that summarizes the incidence of *H. pylori* in East-Africa and its pathology affected by immune responses, genetic factors and environmental factors, including specific regional diets, emphasizing the importance of this topic for East-Africa. In addition, this article reviews effects of the probiotic bacterium LGG on *H. pylori* colonization as well as on inflammation and associated injury of gastric mucosa, in either presence or absence of other treatments. Finally, this review provides substantiation for our on-going probiotic yogurt programs in East-Africa, as reported in this Research Topic in *Frontiers in Microbiology* in another contribution by Westerik et al. (2018), to likely reduce *H. pylori*-associated pathology.

Previous studies also suggested that LGG prevented *H. pylori* colonization, which often occurs in early childhood (Misiewicz et al., 1995; Peña and Versalovic, 2003; Myllyluoma et al., 2008). It is therefore expected that administration of probiotic yogurt containing LGG to children in resource-poor countries from early childhood, can reduce the incidence of *H. pylori* colonization in the general population. Besides prevention of *H. pylori*, LGG may present an approach to establish and manage a harmless relationship between the host and *H. pylori* when the latter one is already present, counteracting the need for *H. pylori* eradication therapy. Kort et al. (2015) and Sybesma et al. (2015) have shown that yogurt containing LRY could be made accessible for people in resource-poor countries in a sustainable way. This is done through providing freeze-dried yogurt starter cultures containing LRY at cost price, in combination with a training program for the local population on standardized and safe yogurt production with locally available equipment.

## Prevalence of *H. pylori* in East Africa

*Helicobacter pylori* is a Gram-negative spiral bacterium that inhabits the surface of the mucous layer of the human stomach and duodenum (upper intestine), usually in a chronic manner, though *H. pylori* colonization can also be temporarily (Hestvik et al., 2010). The majority of carriers have acquired this bacterium through contact with other human beings before the age of 10 years old, more so in cramped housing areas with poor sanitation practices. When present, *H. pylori* is the dominant microorganism, as very few other bacteria can survive in the stomach.

*Helicobacter pylori* is found in more than 50% of world’s population, spread all around the globe (Kodaman et al., 2014). In 10–20% of the carriers, *H. pylori* is believed to be a factor in the development of gastric pathology (Fernando et al., 2001; Gotteland et al., 2006; Kate et al., 2013). Gastric pathology often starts with gastritis (inflammation of the stomach lining), which can become atrophic (degenerating cells) and via gastric metaplasia (abnormal change in tissue) can lead to peptic ulcers and in rare cases to gastric cancer. Duodenal ulcers are much more common than gastric (stomach) ulcers. Over the course of the 20th century, *H. pylori* colonization has become less common in Westernized populations, and in some countries the incidence has dropped below 20% (Go, 2002; Roberts et al., 2016). This is probably associated with improved sanitation, smaller family sizes, and frequent use of antibiotics from childhood (Cover and Blaser, 2009). In contrast, general estimates on the prevalence of *H. pylori* still indicate that over 51% of people living in developing countries, and even 57% of people living on the African continent, are *H. pylori* positive. When taking into account the age of subjects, 52% of African children less than 10 years of age, and over 64% of the African adults are *H. pylori* positive (Zamani et al., 2018). Hence, the high incidence of *H. pylori* in East-Africa might be a risk factor in the development of gastric pathology among the local population. A summary of studies on the incidence of *H. pylori* and gastric pathology in East-Africa can be found in **Table 1**. **Table 1** shows great variety in the incidence rate between 25 and 87%. This may be due to community-specific differences, but also different diagnostic methods used to determine the presence of *H. pylori* (Ricci et al., 2007; Zamani et al., 2018). Notwithstanding, we can conclude that *H. pylori* is a significant risk factor in the development of gastric pathology in East-Africa.

**Table 1:** Summary of studies on the incidence of *H. pylori* and gastric pathology in East Africa.

Year	Country	Study population	Study size	*H. pylori*	Pathogenesis and/or association with *H. pylori*	Comment	Reference
1988	Kenya, rural	Gastritis patients	187	57%	85% of gastritis patients is *H. pylori* carrier	‘Most’ of those who suffered from peptic ulcers or gastric cancer, were *H. pylori* carrier	Lachlan et al., 1988
1981–1986	Rwanda	Endoscopy patients, male/female: 79/94, mean age 35 years	173	75%	29% ulcers of which 100% *H. pylori* carrier 84% gastritis	–	Rouvroy et al., 1987
1993–1995	Uganda	Gastroscopy patients	56	25%	–	*All H. pylori* was found in the antrum (lower part of the stomach) with accompanying chronic atrophic gastritis	Wabinga, 1996
1999	Review of seven African serological studies	Children aged 0–10	635	50%	–		Kidd et al., 1999b
1999	Review of seven African serological studies	Population aged 10–60	1055	61%	Asymptomatic		Kidd et al., 1999b
1999	Review of 21 African endoscopic studies	Dyspepsia patients	3801	72%	92% of gastritis; 92% of duodenal ulcers; and 75% in gastric ulcers patients is *H. pylori* carrier	Duodenal ulcers were four times more common than gastric ulcers. From the dyspeptic subjects suffering from atrophy intestinal metaplasia, on average 77% was found to be carrier of *H. pylori*.	Kidd et al., 1999b
2001	Zambia	General population, male/female: 79/142, >18 years	221	81%	7% gastric lesions 2.6% duodenal ulcers, 0.5% gastric ulcers, and 4% gastritis	35% of the subjects were HIV+, but none of the symptomatic dyspeptic subjects were HIV+, suggesting a protective effect of the HIV virus against pathology of *H. pylori*.	Fernando et al., 2001
2006	Uganda	Cancer patients, male/female: 284/549, age range	833	87%	–		Newton et al., 2006
2010	Uganda	Children in Kampala, male/female: 211/216, mean age 4.8 ± 3.6 years, age range 0-12	427	44%	–	No difference in *H. pylori* colonization between different levels of poverty of the children, as defined by the wealth index. The prevalence rate declined in older children, which might be explained by spontaneous eradication, the use of antibiotics or an increase in antibody production	Hestvik et al., 2010
2006	Kenya	Children 0–3, male/female: 103/92, mean age 17.7 months	195	46%	–		Langat et al., 2006
2014	Uganda	Dyspepsia patients, male/female: 71/96, mean age 48 ± 18 years	167	33%	56% of gastric ulcers and 59% of duodenal ulcers patients is *H. pylori* carrier	35 of the 167 subjects suffered from either gastric or duodenal ulcers	Segamwenge et al., 2014
2014	Uganda	Pregnant women, mean age 24 years, age range 15–42 years	447	45%	–	Women from five different centers from different regions spread over the country were enrolled in the study. The prevalence in the different centers ranged from 18% in a center in Northern Uganda to 61% in a low-income densely populated division of the capital city.	Baingana et al., 2014
2015	Uganda, rural	Patients with gastrointestinal complaints, male/female: 58/116	174	37%	–	The low *H. pylori* carrier rate is explained by the relatively high average age of the subjects, whereby the authors state that *H. pylori* colonization is expected to decrease with age. This is supported by their finding that the highest incidence of *H. pylori* (74%) is in the lowest age bracket (below 21 years) of their study.	Tsongo et al., 2015
2017	Uganda	Symptomatic peptic ulcer patient, male/female: 63/79, mean age 40 ± 18 years, age range 13–85 years	142	22%		The study showed that 29% and 48% of the *H. pylori* samples were resistant against the antibiotics clarithromycin, and fluoroquinolones, respectively	Angol et al., 2017

## Pathogenesis and Other Consequences of *H. pylori* Colonization

The high incidence of *H. pylori* among East African populations *per se* does not necessarily cause increased levels of gastric pathology, as *H. pylori* induced gastric pathology is influenced by genetic factors of the *H. pylori* strain as well as by specific immune responses of the host. At the same time, it has been reported that *H. pylori* might exert beneficial functions in African populations (Blaser and Atherton, 2004; Cover and Blaser, 2009).

In the context of pathology, Blaser and Atherton (2004) distinguish two topographic distributions of *H. pylori* induced gastric inflammation, both having different outcomes. Firstly, inflammation could be in the lower part of the stomach only (antral-gastritis), which via a cascade of cell signaling leads to increased gastric acid output and subsequently increases the risk for duodenal ulcers. Secondly, inflammation of the whole stomach (pan-gastritis) could induce cascades of signaling that lead to reduced acid output and associated increased risk for gastric ulcers. This topographic distribution might be determined by genetic factors of *H. pylori* strains as well as environmental factors.

*Helicobacter pylori* strains exhibit a high level of genetic diversity. Certain genetic characteristics of this bacterium have been associated with increased interactions with the host cells. One of the best documented characteristics is the presence of the pathogenicity island *cagA*, which expresses the effector protein CagA that can be injected into epithelial cells (Crabtree et al., 1991; Bravo et al., 2002). Connected to the presence of *cagA* is the presence of an active VacA protein (Kidd et al., 1999a). All *H. pylori* strains contain the *vacA* gene but with a great variance in nucleotide sequence, and not all expressed VacA proteins have the same functional activities. Active VacA causes alterations in epithelial cells and immune cells due to massive vacuolation (Cover and Blanke, 2005). Strains that both express CagA and active VacA are more likely to cause gastric inflammations and subsequent pathology including peptic ulcers and gastric cancer (Cover and Blaser, 2009). Other authors identified the *dupA* gene as a virulence factor in *H. pylori* strains that is associated with duodenal ulcers (Kate et al., 2013). Immune responses against these virulent factors involve Th1 cells, which paradoxically play a major role in *H. pylori* associated pathogenesis by enhancing inflammation (Blaser and Atherton, 2004).

With regards to the virulence factors, people in developed countries are found to be colonized by almost equal proportions of *cagA*+ and *cagA*- strains (50% *cagA*+), whereas people in developing countries are predominantly colonized by *cagA*+ strains (79% *cagA*+) (Parsonnet et al., 1997). However, it has been suggested that people in developing countries respond to *H. pylori* in a way that is associated with a relatively low risk for pathogenesis (Holcombe, 1992; Mitchell et al., 2009). For example, Segal et al. (2001) found that among people in several African countries, gastric cancer accounts only for 2–3% of all cancers, whereas the international average is 9% (Parkin, 2004). This might be due to modulated immune responses to *H. pylori* as a result of infections with a myriad of gastrointestinal pathogens from early life, which primarily occur in developing countries. Mitchell et al. (2009) quantified the immune response by measuring the IgG1/IgG2 ratio (a marker of the T helper cell response) in Sowetan (South-African), German and Australian symptomatic *H. pylori*-positive subjects. A less pro-inflammatory, IgGI predominant response (IgG1/IgG2 ratio > 1) was observed in 81% of Sowetans, but only in 4.7% of Australians and 4.4% of Germans.

The realization that *H. pylori* has colonized in the gut of mankind for thousands of years (Ghose et al., 2002), creating long-standing dynamic equilibriums, led to the assumption that there must be a mutual beneficial relationship (Blaser and Atherton, 2004; Cover and Blaser, 2009). Kodaman et al. (2014) postulate that *H. pylori* co-evolves with host populations and as a consequence, *H. pylori* is less virulent upon colonization in its ‘natural host’ compared to its colonization in populations from other ethnic origins. The authors reported a strong correlation between *H. pylori* virulence in people of Amerindian origin and the presence of *H. pylori* of African origin and concluded that members of multicultural societies were at higher risk of *H. pylori* associated pathology (Kodaman et al., 2014).

In addition to being harmless in many cases, it has even been proposed that *cagA+ H. pylori* could exert beneficial functions in the body. Over the past decennia, gastroesophageal reflux disease (Vicari et al., 1998), Barrett’s esophagus (Vaezi et al., 2000), esophageal adenocarcinoma (Ye et al., 2004) and dysfunctional responses to common allergens leading to subsequent childhood asthma and allergic disorders (Cover and Blaser, 2009) have become more common in the developed world. Recent research has associated the increase of those diseases with the decrease of *cagA*+ *H. pylori* colonized persons. Mechanisms have been proposed on how the absence of *cagA*+ *H. pylori* induced pan-gastritis could lead to an increase in acid production (Blaser and Atherton, 2004), which in turn could lead to an increased risk of the above mentioned diseases. Indeed, Segal et al. (2001) found that incidence of gastro-oesophageal reflux and its complications were low in black populations where the incidence of *cagA*+ *H. pylori* is high. However, there was no uniform agreement on the topic, as for example Moon et al. (2009) found that there is an increase in reflux disease among *H. pylori* positive children in America. However, considering the differences in topographic distribution of gastritis and subsequent different pathologies (Blaser and Atherton, 2004), the study by Moon et al. (2009) did not necessarily contradict those of others.

Another possible beneficial effect of *H. pylori* is protection against tuberculosis, as a study in West Africa indicated that persons with latent tubercular infections have lower chances of re-activating their infections when they are *H. pylori* positive. This is attributed to the fact that *H. pylori* induces the release of IFN-y, an interferon that activates defenses against different types of infections including tuberculosis (Perry et al., 2010). Finally, *H. pylori* is proposed to play a positive role in body weight regulation: *H. pylori* positive persons produce reduced levels of ghrelin, a weight-regulating hormone that is produced for 60–80% in the stomach. *H. pylori* eradication leads to an increased production of ghrelin and a subsequent increase in body weight (Nwokolo et al., 2003).

## Correlation Between Diet and Lifestyle, and *H. pylori* Associated Pathology

There is evidence for a connection between *H. pylori* and dyspeptic symptoms (suffering from indigestion), as it has been shown that the odds ratio of a person with dyspeptic symptoms being a carrier of *H. pylori* is higher than the *H. pylori* incidence in the general population (Kidd et al., 1999b). Furthermore, *H. pylori* eradication leads to long-term cure in the majority of peptic ulcer patients, whereas the natural relapse rate is 70% (Kate et al., 2013).

However, this argument is not a definite proof for a causal relationship between *H. pylori* and dyspepsia. It has been suggested that genetic predisposition and environmental factors such as diet, smoking, age and the use of non-steroidal anti-inflammatory drugs (NSAIDs) can lead to high gastric acid production, which in turn can lead to gastric metaplasia and subsequent development of duodenal ulcers (Tovey, 2009; Kate et al., 2013). Similarly, the intake of iron supplements has been associated with gastric pathology through the induction of oxidative stress (Naito et al., 1995; Fisher and Naughton, 2004). The positive correlation between *H. pylori* and dyspeptic symptoms is attributed to the fact that treatment of duodenal ulcer with acid-reducing medicine facilitates *H. pylori* colonization in the stomach. Hence, the self-reported high incidence of ulcers in Uganda might be partly a result of lifestyle and dietary habits of the local population.

The hypothesis that *H. pylori* colonization is a result rather than a cause of ulcers, is supported by a retrospective case review of 208 persons, of which 37 were diagnosed with duodenal ulcers. A total number of 32 of the ulcer patients had been suffering from the ulcer for more than 6 months, and all 32 were found to be *H. pylori* positive. Only five patients suffered from ulcers less than 6 months, but all five were found to be *H. pylori* negative (Boulos et al., 2002). Upon colonization, *H. pylori* might produce toxic substances that inhibit the natural healing of the ulcers, which explains why *H. pylori* eradication leads to highly increased chances of long-term cure of ulcers (Tovey, 2009; Kate et al., 2013).

Tovey (2009) emphasizes the role of dietary protective factors. Dietary fiber intake was one of the first factors that was suspected to be negatively associated with the incidence of ulcers. In an intervention study among 42 culturally rice-eating subjects with a history of duodenal ulcers, 21 were changed to a predominantly unrefined wheat diet, whereas the remaining 21 continued the rice diet. Over a period of 5 years, 81% of the subjects in the rice-eating group reported ulcers, versus only 14% in the wheat-eating group (Malhotra, 1978). In a Norwegian study, 73 subjects who recently healed from an ulcer (but by whom *H. pylori* was not necessarily eradicated) were assigned to either a high-fiber diet or a low-fiber diet. After 6 months, ulcers had reoccurred in 80% of the subjects on the low-fiber diet, versus only in 45% of the subjects on high-fiber diet (Rydning et al., 1982).

A case–control study in the United Kingdom compared 78 subjects with duodenal ulcer with 156 matching control subjects (two matches for every ulcer subject). After controlling for total calorie intake, it was found that sugar intake correlated positively and high vegetable fiber intake negatively with ulcers, whereas cereal fibers did not show a specific correlation. After further controlling for smoking, social class and body weight, only the intake of refined sugar remained a significant factor that correlated with ulcer incidence (Katschinski et al., 1990).

From an anecdotal study on the incidence of peptic ulcers in sub-Saharan Africa, a correlation between areas with a high incidence of ulcers and high intake of starchy foods, such as bananas, cassava, sweet potato, white wheat flour, white maize flour, and white rice has been suggested. This was opposed to regions with high intake of millet and home-pounded (unrefined) maize, where the incidence of ulcers was low (Tovey, 2009). However, Tovey (2009) suggests that it is not the fiber from the unrefined cereal in itself, but rather the phospholipids, sterol esters, and sterol fractions of the lipid components from the cereal fiber fraction that exercises the protective action.

Other studies identified the plant-derived polyunsaturated fatty acids lipid fraction, such as linoleic and linolenic acid, to have inhibitory effects on *H. pylori*. *In vitro* studies with these components showed to cause cell death to *H. pylori* by damaging the bacterial outer lipid membrane (Thompson et al., 1994). Besides, certain fatty acids are precursors for prostaglandins which protect the gastric mucosa against injury through increased mucus secretion. Furthermore, arachidonic acid has been shown to improve gastric blood vessel synthesis, thereby speeding up gastric healing processes (Hollander and Tarnawski, 1990).

Also in more dated review studies on the relationship between diet and duodenal ulcers Misciagna et al. (2000) concluded that there were strong indications for a negative correlation between duodenal ulcers and the intake of fiber, mainly soluble fiber from fruit and vegetables, and perhaps polyunsaturated fatty-acids, vitamin A, and vitamin C. However, the study made note of the overall poor quality from the studies that led to this conclusion.

Other studies suggest a correlation between intake of salt and gastric pathology, partly modulated through *H. pylori*, most commonly in the form of gastric cancer (Wang et al., 2009). Ulceration has also been mentioned as an outcome of high salt intake (Kato et al., 1992), though another study found no effect of salt intake on gastric inflammation (Lee et al., 2014). An *in vitro* study suggested that salt induces expression of virulence factors in *H. pylori* (Xu et al., 2011), which provides a possible explanation for increased gastric pathology upon high salt intake.

## The Probiotic LGG in *H. pylori* Eradication Therapy

One of the few other bacteria that can abide under the same gastric circumstances as *H. pylori* (Bezkorovainy, 2001), being resistant to acid and bile (Fernandez et al., 2003), is LGG and its generic variant LRY. The probiotic bacterium LGG is world’s best documented probiotic bacterium, with many unique characteristics and reported health benefits (Segers and Lebeer, 2014). Especially to provide access to this bacterium for people in developing countries, the LRY has been incorporated in an affordable probiotic starter culture that enables local communities to make their own probiotic fermented food (Kort et al., 2015; Westerik et al., 2016). *H. pylori* can be inhibited in a dose dependent manner by LRY through several pathways, as discussed below, and hence may play a role in the reduction of *H. pylori* related gastric pathology in East Africa (Angol et al., 2017).

Currently, the standard method to eradicate *H. pylori* is a three-component therapy treatment that combines acid suppression with two antibiotics for 1 week. This method is also used in East Africa for those who can afford it (Angol et al., 2017). However, *H. pylori* becomes increasingly resistant against this therapy, and the success rates are dropping below 70% (Malfertheiner et al., 2012). Furthermore, the therapy typically has low patient compliance due to many side effects such as diarrhea, nausea, vomiting, bloating, and abdominal pain (Armuzzi et al., 2001a). The European *Helicobacter* Study Group recently acknowledged that certain probiotics show promising results as an adjuvant treatment in reducing side effects of the antibiotic therapy (Malfertheiner et al., 2012).

A summary of studies on the role of LGG in *H. pylori* eradication therapy can be found in **Table 2**. All studies confirmed a beneficial effect of LGG in reducing side effects of eradication therapy. Five studies with an average sample size of 70, showed no significant effect on the *H. pylori* eradication rate. However, the sixth study with a sample size of 650 showed *H. pylori* eradication rates of 87% and 73% in the LGG-supplemented group and the control group, respectively.

**Table 2:** Summary of studies on the role of LGG in *H. pylori* eradication therapy.

Sample size	Population	LGG treatment	Mode of administration	Symptoms that have reduced incidence during the treatment week (week 1)	Effect on eradication rate	Study type	Comment	Reference
120	Italian, male/female: 54/66, mean age 37 ± 11 years	6 × 10^9^ cfu twice daily for 14 days	Freeze-dried powder, mixed with water	Taste disturbance 7% vs. 27%, bloating 17% vs. 40%, diarrhea 7% vs. 23% for the treatment group and the control group, respectively	No effect	Non-placebo controlled	In the week after the treatment, the differences remained significant. In the second and the third week after the therapy most side effects faded out in both groups, only bloating remained to have a high incidence, with 7% vs. 17% in the third week.	Armuzzi et al., 2001b
60	Italian, male/female: 25/35, mean age 40 ± 12 years	6 × 10^9^ cfu twice daily for 14 days	Freeze-dried powder, mixed with water	Taste disturbance 23% vs. 50%, diarrhea 3% vs. 27%, nausea 10% vs. 37% for the treatment and the placebo group, respectively	No effect	Double-blind placebo controlled	Differences slowly faded out after 2 weeks. Bloating showed confusing results. In the first week the incidence was 37% vs. 57%, but in subsequent weeks no significant differences were observed.	Armuzzi et al., 2001a
42	Italian, male/female: 19/23, age range 18–61 years	6 × 10^9^ cfu LGG twice daily for 14 days	Freeze-dried powder	Taste disturbance 10% vs. 40%, diarrhea 5% vs. 30% for the treatment and the placebo group, respectively	No effect	Triple-blind placebo controlled	–	Cremonini et al., 2002
47	Finnish, male/female: 18/29, mean age 56 years, age range 24–96 years	6 × 10^8^ cfu twice daily for 7 days, once daily for another 3 weeks	Milk-based fruit drink also containing *L. rhamnosus* LC; *P. freudenreichii* ssp. *shermanii* JS; and *B. breve* Bb99	Incidence of side effects is the same, severity of side effects decreases	No effect	Placebo-controlled, double-blind randomized pilot study	The study included analyses of fecal samples, from which was concluded that despite the severe antibiotic treatment, LRY concentrations increased in the probiotic groups, though the levels came back to baseline 6 weeks after the intervention.	Myllyluoma et al., 2005
83	Polish children 5–17 years	10^9^ cfu LGG twice daily for 7 days	Freeze-dried powder	Decrease in diarrhea from 6% vs. 20% between in the treatment and control group, respectively	No effect	Double-blind placebo-controlled	Outcomes on diarrhea reduction not statistically significant.	Szajewska et al., 2009
650	Croatian, male/female: 283/367, mean age 52 ± 11 years	10^8^–10^10^ LGG twice daily for 14 days	Capsule also containing *Bifidobacterium*	Epigastric pain, bloating, flatulence, taste disturbance, nausea, heartburn and a strong reduction of diarrhea from 18% in the placebo group to 4% in the treatment group	Increased to 87% vs. 73% in the placebo	Prospective, randomized, placebo-controlled, double-blind, multicenter trial		Hauser et al., 2015

## Ulcer Prevention and Suppression by LGG

Apart from its role as co-supplement during *H. pylori* eradication therapy, LGG has a direct inhibitory effect on *H. pylori* and has the potential to directly prevent and reverse gastric pathology in East African populations. Accordingly, in a review of 13 clinical trials reporting on the activity of probiotics on *H. pylori*, Hamilton-Miller (2003) concludes that probiotics can reduce the severity of *H. pylori* induced pathology, but are not able to eradicate *H. pylori* completely. Three distinct pathways in which LGG counteracts *H. pylori*-induced gastric pathology in the stomach and duodenum can be discriminated: (i) competition for binding sites between LGG and *H. pylori*, (ii) attenuation of the host’s *H. pylori*-induced apoptosis, inflammation responses and stimulation of angiogenesis, and (iii) production of anti-microbial substances such as lactic acid.

### Competition for Binding Sites

**FIGURE 1:**
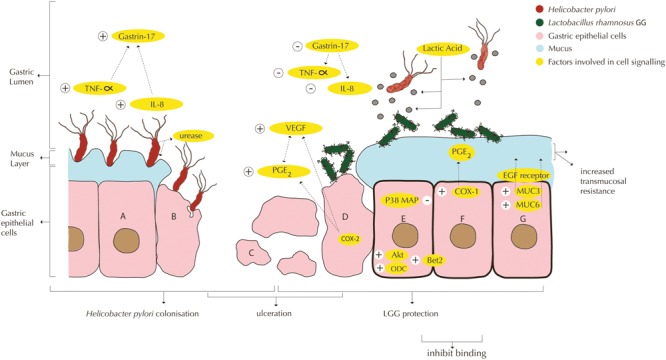
Interactions of *Lactobacillus rhamnosus* GG or LRY (green) with *Helicobacter pylori* (red) and damaged epithelial cells at the gastric mucosal surface. (A) *H. pylori* upregulates TNF-α (Peña and Versalovic, 2003) and IL-8 (Myllyluoma et al., 2008; Rokka et al., 2008), which in turn upregulate gastrin-17 (Myllyluoma et al., 2007). *H. pylori* produces urease to reduce the pH in its immediate environment as means of survival (Chen et al., 2010). (B) Interactions under A are causing inflammation and apoptosis. (C) Gastric inflammation and apoptosis lead to ulceration. (D) LRY binds specifically at affected (mucosal) tissue due to change in microbiota and inflammatory markers (immunoglobulins and cytokines) and qualitative and quantitative changes in the mucus (Lam et al., 2007b). LRY upregulates COX-2 protein expression in damaged (ulcerated) stomachs (Jackson et al., 2000), which induces PGE_2_ modulated vascular endothelial growth factor (VEGF) expression, causing angiogenesis (Korhonen et al., 2004; Tamura et al., 2006; Lam et al., 2007b). LRY inhibits *H pylori* induced IL-8 (interleukin-8) release (Myllyluoma et al., 2008; Rokka et al., 2008) and TNF-α release (Peña and Versalovic, 2003; Kim et al., 2006). Through suppression of TNF-α and IL-8, *H pylori* induced elevated gastrin-17 levels are attenuated (Myllyluoma et al., 2007). (E) LRY inhibits *H pylori* adhesion (Myllyluoma et al., 2008), which appears to be related to competition for binding sites (Peña and Versalovic, 2003; Myllyluoma et al., 2008). LRY activates Akt/protein kinase B, which is an anti-apoptotic signaling pathway (Yan and Polk, 2002). LRY blocks p38 MAP, which is a pro-apoptosis (cell survival) signaling pathway (Yan and Polk, 2002). LRY induces upregulation of ornithine decarboxylase (ODC) (enzyme essential in DNA stabilization and thus cell growth) and B-cell lymphoma 2 (Bcl-2) causing attenuation of apoptosis (Lam et al., 2007b). LRY shows antagonistic activity against *H. pylori in vitro*, possibly associated with lactic acid production (De Keersmaecker et al., 2006; Hütt et al., 2006). Lactic acid increases the cell wand permeability of *H. pylori*. Besides, production of exopolysaccharide (Allonsius et al., 2017), lectin (Petrova et al., 2016), and/or peptides with antimicrobial activity (Lu et al., 2009) by LRY has been shown to inhibit other pathogenic bacteria, however, this effect has not been shown for *H. pylori* specifically. LRY inhibits urease activity of *H. pylori* (Chen et al., 2010). (F) LRY upregulates COX-1 (cyclooxygenase-1) protein expression (Jackson et al., 2000; Lam et al., 2007a) in normal stomachs (ulcer prevention), which increases PGE_2_, which in turn protects mucus cells and increases trans mucosal resistance, thereby protecting mucosal cells from apoptosis (Jackson et al., 2000; Lam et al., 2007a). LRY upregulates the expression of MUC3 and MUC6 mucin gene, causing significant increase in thickness of the basal mucus layer (Mack et al., 2003; Lam et al., 2007a). (G) LRY causes upregulation of phosphorylation level of epidermal growth factor receptor (EGF receptor), leading to cell proliferation and angiogenesis and suppresses cell apoptosis (Lam et al., 2007b). LRY initially improves epithelial (mucosal) barrier function (Gotteland et al., 2001; Myllyluoma et al., 2008) but increases *H. pylori* induced barrier deterioration after incubation for 24–42 h, hence causes delayed cell membrane damage and leakage (Myllyluoma et al., 2008).

*In vitro* pre-treatment of epithelial glandular cells (coca-2 cell culture) with 10^7^ cfu/ml LGG was found to inhibit subsequent adhesion by *H. pylori* with 53%, and at 10^9^ cfu/ml LGG inhibited *H. pylori* with 66%. Competition for binding sites on the epithelial cells was cited as a probable cause (Myllyluoma et al., 2008) (**Figure 1**), which appeared to be unrelated to adhesion capacity or organic acid production. Note that binding alone is not a marker for anti-*H. pylori* activity (Peña and Versalovic, 2003; Myllyluoma et al., 2008).

Secondly, a study on mice found that during a pre-treatment with a combination of the probiotic bacteria *L. acidophilus* R0052 and *L. rhamnosus* R0011 and subsequent administration of *H. pylori*, colonization was reduced from 100% in the control group to 50% in the probiotic pre-treated group (Johnson-Henry et al., 2004). Note that this effect has not been proven for LGG specifically.

In a third study, a decrease in *H. pylori* of 27% was measured in a probiotic intervention among 13 Finnish *H. pylori* positive subjects, based on the well accepted ^13^C-Urea Breath Test, which values were an indirect indicator of *H. pylori* density in gastric mucosa. The subjects daily consumed a drink with four probiotic strains, including LGG, with each of the strains in a concentration of 2.5 × 10^9^ cfu, for the duration of 56 days, for the duration of 56 days (Myllyluoma et al., 2007).

Upon the formation of an ulcer, probably regardless of the causative factor of the ulceration, a spectrum of bacteria other than *H. pylori* has been found to colonize the ulcer surface and to subsequently impair ulcer healing (Elliott et al., 1998). This has been attributed to a local increase of the pH around the ulcer surface, allowing for strongly increased bacterial growth as compared to normal stomach tissue. The same study found that antibiotic treatment, preferably with a mixture of different antibiotics, increased ulcer healing. Secondly, promotion of lactic acid bacteria (non-specified) in the stomach competed with outgrowth of other possibly harmful bacteria, and increased the rate of ulcer healing. Other studies have indicated specifically the suppressing effect of LGG on several pathogens (Rinkinen et al., 2003; Hütt et al., 2006) and its site-specific binding to damaged tissue (Ouwehand et al., 2003).

### Modulation of Immune Responses

LGG has shown to modulate local immune responses upon colonization with *H. pylori* or ulceration through the pathways as summarized in **Figure 1**.

*In vitro* pre-treatment of epithelial glandular cells (coca-2 cell culture) with 10^7^ cfu/ml LGG was shown to counteract acute *H. pylori* cell membrane leakage by initially tightening the barrier function (Gotteland et al., 2001; Myllyluoma et al., 2008). However, over time *H. pylori* induced barrier deterioration after incubation for 24–42 h, causing delayed cell membrane damage and leakage (Myllyluoma et al., 2008).

*H. pylori* upregulates TNF-α (Peña and Versalovic, 2003) and IL-8 (Myllyluoma et al., 2008; Rokka et al., 2008), which in turn upregulate gastrin-17. IL-8 is a chemokine which induces inflammation (Rokka et al., 2008) and TNF-α is a cytokine which induces apoptosis (Peña and Versalovic, 2003). Gastric inflammation and apoptosis lead to ulceration. Gastrin-17 has been associated with gastric cancer (Myllyluoma et al., 2007). LGG inhibits *H pylori* induced IL-8 (interleukin-8) release (Myllyluoma et al., 2008; Rokka et al., 2008) and TNF-α release (Peña and Versalovic, 2003; Kim et al., 2006), although it must be noted that another *in vitro* study (Zhang et al., 2005) showed that high doses of LGG (10^10^ cfu) can increase IL-8 production in the absence of *H. pylori*. Through suppression of TNF-α and IL-8, *H pylori* induced elevated gastrin-17 levels were attenuated (Myllyluoma et al., 2007). A decrease in the hormone gastrin-17 levels was measured in subjects who daily consumed a drink with four probiotic strains including LGG. This study concluded that gastrin-17 could be seen as a maker for non-atrophic gastritis, that probiotics can have a moderate positive influence on non-atrophic gastritis (Myllyluoma et al., 2007).

Apart from *H. pylori*, alcohol has been associated with gastric mucosal damage, and alcoholism can be another cause of peptic ulcers. Rats pre-treated with LGG for 3 days responded to the administration of ethanol in a dose-dependent manner: 1 h after administering 10 ml/kg bodyweight of 60% v/v ethanol, the 2 × 10^8^ cfu/day pre-treated group did not show any difference, while in the 2 × 10^9^ cfu/day group showed 45% smaller gastric lesions as compared to the control group (Lam et al., 2007a). The study concluded that LGG significantly increases the mucosal layer and mucosal integrity (trans mucosal resistance) through upregulated expression of MUC3 (Mack et al., 2003) and MUC6 mucin genes, thereby counteracting the effects that ethanol normally has on the mucus layer (Lam et al., 2007a). Nevertheless, LGG regulates COX-1 (cyclooxygenase-1) protein expression (Jackson et al., 2000; Lam et al., 2007a) in normal stomachs, which increases PGE_2_, which protects mucus cells and increases trans mucosal resistance, thereby protecting mucosal cells from apoptosis (Jackson et al., 2000; Lam et al., 2007a) and reducing chances of ulceration.

In a subsequent study of Lam et al. (2007b), ulcers were induced by luminal application of acetic acid solution, and LGG was administered in the same manner as described for the previous study, but this time after the ulcer-induction. LGG supplementation had no obvious effects in the control group, but for the ulcer-induced group the larger dose at 10^9^ cfu/day induced enhanced cell proliferation of 54%, increased blood vessels generation (angiogenesis) by 41% at the ulcer margins and reduced cell death (apoptosis) by 33%, thereby obtaining significantly reduction of gastric ulcer area by 32% after 3 days of LGG administration. These effects were found to be modulated firstly by the upregulation of phosphorylation level of epidermal growth factor receptor (EGF receptor) causing angiogenesis, cell proliferation and attenuation of apoptosis. Secondly, COX-2 protein expression was upregulated, which induces PGE_2_ modulated vascular endothelial growth factor (VEGF) expression, causing angiogenesis (Korhonen et al., 2004; Tamura et al., 2006; Lam et al., 2007b). Thirdly, LGG upregulates ornithine decarboxylase (ODC) (enzyme essential in DNA stabilization and thus cell growth) and B-cell lymphoma 2 (Bcl-2), thereby causing attenuation of apoptosis (Lam et al., 2007b). The healing continued upon administration of LRY for more days. The study concludes that LGG does not affect the normal gastric mucosa, but normalizes gastric mucosa that is altered by events such as ulcers. LGG has shown to bind specifically at affected (mucosal) tissue due to a change in microbiota and inflammatory markers (immunoglobulins and cytokines) and qualitative and quantitative changes in the mucus. The study has not shown whether it is the live LGG, its metabolites, its cell wall components or other results of gene expression that exert the healing properties of the organism (Lam et al., 2007b).

Similarly, in an intervention among 16 human subjects, a 5-day pre-treatment with a probiotic dairy product containing among others 2.4 × 10^9^ cfu LGG per day, has been proven to stabilize the intestinal barrier function against increased permeability normally induced by NSAIDs by 77%, thereby preventing the alterations from future pathology such as ulcers (Gotteland et al., 2001). Heat-killed LGG did not show protective effects. Interestingly, a study by Kamil et al. (2007) on ulceration in the small intestine of rats as induced by NSAIDs, found that although LGG increased cell proliferation and reduced cell apoptosis, it still aggravated the NSAID induced ulcer size, possibly through increased inflammation.

Other pathways include the activation of Akt/protein kinase B by LGG, which is an anti-apoptotic signaling pathway (Yan and Polk, 2002). Secondly, LGG blocks p38 MAP, which is a pro-apoptosis (cell survival) signaling pathway (Yan and Polk, 2002). Lastly, *H. pylori* produces urease to reduce the pH in its immediate environment as means of survival, which is inhibited by LGG (Chen et al., 2010), thereby reducing its chances for survival.

### The Probiotic LGG Produces Antimicrobial Substances Against *H. pylori*

Antagonistic activity against *H. pylori in vitro* has been associated with lactic acid production (De Keersmaecker et al., 2006; Hütt et al., 2006; Segers and Lebeer, 2014) and to a lesser extent with the production of other short-chain fatty acids (SCFAs) (Gotteland et al., 2006). Lactic acid increases the cell wall permeability of *H. pylori* (**Figure 1**). Besides, production of exopolysaccharide (Allonsius et al., 2017), lectin (Petrova et al., 2016), and/or peptides with antimicrobial activity (Lu et al., 2009) by LGG has been shown to inhibit other pathogenic bacteria, however, this effect has not been shown for *H. pylori* specifically. A summary of studies on the effect of LGG on *H. pylori* and gastric pathology can be found in **Table 3**.

**Table 3:** Summary of studies on the effect of LGG on *H. pylori* and gastric pathology.

Study sample type	Pre-treatment	Treatment	Mode of LGG administration	Effect	Reference
*In vitro* coca-2 cell culture	LGG	*H. pylori*	Cells inoculated with LGG suspended in DMEM medium	Inhibition of *H. pylori* adhesion by max 66%	Myllyluoma et al., 2008
Mice	*L. rhamnosus* R0011 and *Lactobacillus acidophilus* R0052	*H. pylori*	Powder dissolved in sterile distilled water, orally administered.	*H. pylori* colonized 50% of the treatment group vs. 100% in the control group	Johnson-Henry et al., 2004
13 human subjects	Subjects are *H. pylori* positive	LGG, 2.5 × 10^9^ daily for 54 days	Probiotic drink also containing *L. rhamnosus* LC; *P. freudenreichii* ssp. *shermanii* JS; and *B. breve* Bb99	Decrease of *H. pylori* colonization by 27% measured by 13C-UBT	Myllyluoma et al., 2007
Rats	LGG 1 × 10^8^ or 1 × 10^9^ cfu twice daily for 3 days	Ethanol (inducing ulcers)	Culture suspended in sterilized water	Treatment group 45% smaller lesions	Lam et al., 2007a
Rats	Acetic acid solution (inducing ulcers)	LGG 1 × 10^8^ or 1 × 10^9^ cfu twice daily for 3 days	Culture suspended in sterilized water	Reduced gastric ulcer area by 32%	Lam et al., 2007b
16 human subjects	LGG 1 × 10^7^ trice daily for 5 days	NSAIDS	Commercial dairy product also containing *L. helveticus* and *L. acidophilus*	Reduced gastric permeability by 77% as compared to the non-treatment group	Gotteland et al., 2001
Rats	Acetic acid solution (inducing ulcers)	Lactulose to stimulate growth of LAB	Lactulose dissolved in drinking water	Significant reduction of pathogen colonization, significant increased ulcer healing	Elliott et al., 1998

## A Sustainable Nutritional Intervention in East Africa – the Case Study of Uganda

The absence of dietary protective factors in the traditional starch-based diet in Uganda which mainly consists of bananas, roots and refined cereals, might be a major factor causing the high incidence of ulcers in this country. In rural Uganda, when one experiences dull, sharp or burning pain in the upper abdomen that might indicates dyspepsia, subsequent self-medication with simple and cheap acid suppressors (magnesium trisilicate) is the common practice. Many clinics lack any form of diagnostic equipment and will diagnose ulcers based on description of symptoms only, and subsequently mainly prescribe acid suppressors, though other medicine like histamine antagonists (H2 blockers) and proton-pump inhibitors that suppress acid secretion are also common. The frequent advice is to not use NSAIDs, spices, cigarettes, alcohol or carbonated drinks. Some clinics can perform serum-blood test to detect the presence of *H. pylori* antibodies. Only the minority of the population that enjoys a better economic status might visit private clinics that can perform endoscopy, and might opt for the more expensive eradication therapy. In its clinical guidelines, the Uganda ministry of health advises to primarily treat ulcers with acid suppressors and encourages regular, small and frequent meals, as well as the consumption of milk (Ministry of Health, 2010).

Kort and Sybesma (2012) and Kort et al. (2015) described an intervention with the generic variant of LGG, in form of an LRY containing yogurt drink, which is locally produced (Westerik et al., 2016) and subsequently consumed by resource-poor communities in rural Uganda. We propose such an intervention as a preferred option to alleviate the burden of *H. pylori* induced pathology in resource poor communities. Blaser and Atherton (2004) mentioned that a beneficial function of *H. pylori* colonization is the reduction of childhood diarrhea. However, when *H. pylori* is in part replaced by LRY, this beneficial function of *H. pylori* might not be lost: LRY has the proven ability to reduce certain types diarrhea (De Roos and Katan, 2000; Szajewska et al., 2007; Allen et al., 2010; Guandalini, 2011; Guarino et al., 2015), but without the adverse risks that are associated with *H. pylori* colonization. The local production of probiotic fermented foods can be extended to cereal fermentations, most importantly as a variation on the already popular fermented millet drink (obushera) for the case of Uganda (Westerik et al., 2016). As it has been suggested that cereal fibers provide additional protection against gastric pathology (Malhotra, 1978; Tovey, 2009), this high-fiber drink may exhibit dual protective action against ulcer formation.

## Conclusion

Studies on the incidence of *H pylori* in East Africa showed widely varying outcomes, ranging between 25 and 87% in various population groups, possibly due to different detection methods used, or differences in study population. Pathology upon *H. pylori* colonization is modulated by several factors including the presence of virulence factors in the *H. pylori* strain, the ‘ethnic’ origin of the strain, and the specific immune responses of the host. Apart from *H. pylori* colonization, gastric pathology including ulceration is also affected by lifestyle factors, including diet. The right dietary factors have been shown to directly inhibit *H. pylori* as well as reduce *H. pylori* induced pathology.

It is expected that administering probiotic yogurt to children in developing countries from early childhood can reduce the incidence of *H. pylori* colonization in the general population. Besides prevention of *H. pylori*, LGG or its generic variant LRY may present an approach to establish and manage a harmless relationship between the host and *H. pylori* when the latter one is already present, counteracting the need for *H. pylori* eradication therapy. This alternative approach is cheaper and does not carry the risk of extensive antibiotic resistance (Michetti, 2001), and is feasible to be implemented sustainably through locally produced yogurt containing LRY.

It should be noted that major changes in diet might play an equally important role in the prevention and relieve of gastric pathology. In Uganda, locally available foods with protective factors include unrefined wheat, unrefined maize, unrefined rice, millet, soy beans, full-cream milk, spinach, and cabbage (Tovey, 2009). However, consumption of these products would require education of the population and a change in attitude, since even the rural population as a rule brings produced cereals to an electrical mill, in which the bran is separated from the cereal and subsequently the bran fraction is being used solely for animal feeds. An intervention with the mentioned locally produced yogurt could capture the dietary protective benefits of milk as well as the those of LRY.

## Author Contributions

NW reviewed the cited literature and drafted the manuscript under guidance of RK. WS, GR, and RK critically read and corrected the draft versions of the article.

## Conflict of Interest Statement

RK and WS are co-founders of the Yoba for Life Foundation (2009), a non-profit organization, accredited by the Dutch Tax Authorities as a Public Benevolent Institution (PBI), which aims to promote local production and consumption of fermented products in Africa. NW is the Country Coordinator of the Yoba for Life Foundation in Uganda. African fermented products made with the Yoba starter culture, are not marketed by the foundation as such, but the Yoba for Life Foundation stimulates local production and ownership, allowing income-generating activities for African small-scale entrepreneurs in the food sector. The Yoba for Life Foundation distributes and sells ready-to-use sachets with dried bacterial starter cultures at cost price, through a network of partners and volunteers to facilitate the local production of dairy and cereal-based products by controlled bacterial fermentation. The Yoba starter culture contains *Lactobacillus rhamnosus* yoba 2012, which is a generic variant of *Lactobacillus rhamnosus* GG. The remaining author declares that the research was conducted in the absence of any commercial or financial relationships that could be construed as a potential conflict of interest.
